# Prevalence of hypodontia and supernumerary teeth in a German cleft lip with/without palate population

**DOI:** 10.1186/s12903-021-01420-7

**Published:** 2021-02-11

**Authors:** Liesa Heidi Möller, Winnie Pradel, Tomasz Gedrange, Ute Ulrike Botzenhart

**Affiliations:** 1 Department of Orthodontics, Carl Gustav Carus Campus TU Dresden, Fetscherstrasse 74, Haus 28, 01307 Dresden, Germany; 2 Department of Oral and Maxillofacial Surgery, Dresden University Hospital, Fetscherstrasse 74, 01307 Dresden, Germany

**Keywords:** Hypodontia, Supernumerary teeth, Cleft lip, Cleft palate

## Abstract

**Background:**

The distribution of dental abnormalities among cleft patients concerning cleft type frequently poses ambiguity wherefore the aim of this study was to evaluate the prevalence of hypodontia and supernumerary teeth in an exemplary German cleft population dependent on the cleft type.

**Methods:**

Radiographs and dental records of cleft patients, which had been treated and followed up in the Department of Oral and Maxillofacial Surgery, University Hospital Carl Gustav Carus Campus, Dresden, Germany (investigation period of 22 years) were evaluated concerning hypodontia and supernumerary teeth dependent on the cleft type. Out of 386 records, 108 patients met the inclusion criteria: non-syndromic cleft of the alveolus with or without palate (CL/P), at least one clear panoramic x-ray, sufficient dental records. Statistical analysis was performed using x-square and binominal test (p ≤ 0.05).

**Results:**

Hypodontia was more frequent (54/50%) than supernumerary teeth (36/33.3%) and was more common in bilateral clefts of the lip and palate (BCLP) (70.1%) than in unilateral clefts of the lip and palate (UCLP) (51.6%) or clefts of the lip and alveolus (CLA) (34.5%) (p << 0.001). There was an average of 0.9 missing teeth per patient, thereof the upper lateral incisor was most often affected (23.2%). In contrast, supernumerary teeth were more frequent in CLA (51.7%; p = 0.014) than UCLP (29.0%) and BCLP patients (17.6%).

**Conclusion:**

The prevalence for numerical dental anomalies was significantly different among the cleft types. Hypodontia significantly increased with the extend of the cleft, whereas the prevalence of supernumerary teeth decreased.

## Background

Clefts of the lip with or without palate (CL/P) are one of the most common craniofacial malformations [1, 2]. The monitoring of malformations in Saxony-Anhalt, a member of ICBDSR (International Clearinghouse for Birth Defect Surveillance and Research) listed a basic prevalence over the past 12 years of 1.32 per 1.000 live births with CL/P in Germany [3].

These deformities often go along with dental abnormalities in number, shape, location and time of eruption [4, 5]. In affected persons the development of the dentition is disturbed [6], with hypodontia and supernumerary teeth to be found in a much higher frequency than in a healthy European population. The missfusion of the epithelium in the region of the cleft can either result in an additional tooth, in cases of less mesenchyme, in a microform to develop or even hypodontia of the lateral incisive [7]. Others discuss the environmental impact of the surgical closure of the hard palate in early childhood to be responsible for the loss of tooth germs of the permanent second premolars [8]. Besides these environmental impacts there are different genes and gene-loci described, such as MSX1 and PAX9, causing the combined development of orofacial clefts and hypodontia [9].

In the healthy European population a calculated prevalence of 5.3–5.6% can be found for hypodontia [10], but in cleft patients in many studies the prevalence is reported to be much higher, ranging from 36.0–77.0% [7, 11–13]. In contrast supernumerary teeth occur with a prevalence of about 1.0–2.2% in a European non-cleft population [14], whereas a prevalence of 4.6–42.0% can be found for supernumerary teeth in different cleft-groups [7, 11, 13, 15]. This variation can be explained by different sample composition and ethnic origin of the CL/P individuals. Cleft patients often need a combined and complementary treatment including oral and maxillofacial surgery, otorhinolaryngology, speech therapy and orthodontics. Especially for orthodontists and surgeons, local data of tooth buds and the development of the dentition are very interesting and helpful to set time and place for interventions and facilitate treatment planning.

*The aim of the present study* was to evaluate the prevalence and distribution of hypodontia and supernumerary teeth in association with gender, cleft side and cleft type in a local exemplary German non-syndromic CL/P population.

## Methods

This epidemiological and retrospective study was carried out in the Department of Orthodontics, Medical Faculty Carl Gustav Carus Campus, Dresden, Germany according to the World Medical Association Declaration of Helsinki and approved by the ethical review committee, TU Dresden, Germany with the number: EK 442122014.

Data of the present study were obtained from records of the pool of cleft patients, which had been treated and followed up in the Department of Oral and Maxillofacial Surgery, University Hospital Carl Gustav Carus Campus, Dresden, Germany—one of three cleft centers in Saxony, Germany—between January 1994 and November 2016. It predominantly consisted of Caucasian male and female patients, all of whom aged six years or older (ranging from 6 years and 0 months to 18 years and 8 months; average age of patients at the time of x-ray diagnosis was 9 years and 4 months ± 2 years and 11 months).

The inclusion criteria of this study were: male and female patients aged six years or older, diagnosed with cleft lip and alveolus (CLA) or cleft lip and palate (CLP), without any coexisting genetically syndromes and at least one analyzable orthopantomogram. Patients with an isolated cleft of the lip and/ or the palate as well as patients with unique, atypical types of clefts were excluded from this study. Children younger than six years of age were also excluded from the study due to the possible inaccuracy while identifying hypodontia and supernumerary teeth, especially of second premolars, in a radiograph at this age [16, 17].

A hundred and eight patients out of 386 CL/P individuals met the inclusion criteria. For detailed information about the inclusion and exclusion process, see Table 1.

**Table 1:** Analyzed cleft collective and the number of included individuals

Total number of cleft patients		386
Exclusion criteria	Isolated cleft lip	42
	Isolated cleft palate	139
	Atypical type of cleft	11
	Coexisting syndrome	12
	Unclear panoramic x-ray	74
Total number of patients included in the study		108

All records were investigated using at least one clear panoramic x-ray of each patient. If available, in addition, initial and follow-up radiographs were checked for dental anomalies to eliminate ambiguity and ensure results. Results were double-checked and compared with findings of dental records and intraoral photographs concerning extractions, for example by the general dentist. Tooth counts and percentages were used to describe hypodontia and supernumerary teeth excluding the third molar region (due to comparability with other studies and the typical development of these tooth buds beginning at an age of 7–9 years [18] and the resulting inaccuracy of diagnosing hypodontia of third molars at the chosen minimum age of 6 years).

To avoid different interpretations due to personal examination all panoramic x-rays were observed by one single examiner. In case of inconsistency, difficult or unclear findings these were discussed with another observer. If no agreement could be reached, the patient was excluded from the study.

For grouping and statistical analysing, the cleft sample was divided into three groups typified by cleft type, representing different grades of severity: CLA, unilateral CLP (UCLP) and bilateral CLP (BCLP). All CLA patients showed an unilateral cleft type. The exact cleft classification was registered by checking the dental records of the included patients. In the Department of Oral and Maxillofacial Surgery the “LAHS-Code”, as it has been described by Koch (1968), is used as gold standard to classify the cleft type. For detailed information on the distribution of cleft groups, see Table 2.

**Table 2:** Distribution of analyzed cleft patients according to cleft type, cleft side and gender

	CLA				UCLP			BCLP
Cleft side	Right	Left	Bilateral	Total	Right	Left	Total	Total
Male	6	10	0	16	12	29	41	12
Female	3	10	0	13	10	11	21	5
Total	9	20	0	29	22	40	62	17

CLA, cleft lip and alveolus; UCLP, unilateral cleft lip and palate; BCLP, bilateral cleft lip and palate

Statistical analysis was performed using MATLAB (version 1.8.0_121(R2017b). Natick, Massachusetts: The MathWorks Inc.). Statistical significance was calculated using Chi-square and binominal tests with significance level set at p ≤ 0.05

## Results

Two hundred and three out of the 386 patients analyzed had to be excluded for medical reasons, as there had been a syndromic cleft background, or a cleft other than CL/P. Seventy-four individuals could not be included because of unclear or non-existing radiographic diagnostics (see Table 1).

The final cleft sample consisted of 108 patients, 69 males and 39 females corresponding to a gender distribution of 1.77:1 male to female. Ninety-one patients showed unilateral clefts (all CLA and UCLP patients), 31 right-sided and 60 left-sided clefts (see Table 2).

### Hypodontia

In our study, a total of one hundred and two missing teeth could be found within the cleft collective analyzed, affecting 54 out of 108 patients (50%), thereof 33 male (47.8%) and 21 female (53.8%) with no gender dependency to be found (p = 0.548). The upper lateral incisor was the most commonly missing tooth (23.1%, 50 out of 216 possible teeth), followed by the maxillary second premolar (14.4%) and the mandibular second premolar (5.1%). Hypodontia exclusively occurred in the maxilla in a frequency of 81.5%, in both jaws in a frequency of 15.8% and exclusively in the mandible in a frequency of 3.7% of the patients. Furthermore, hypodontia was significantly depending on the severity of the cleft (p << 0.001). In 34.5% of CLA patients, at least one tooth was missing, whereas UCLP and BCLP patients were affected by hypodontia in 51.6% and 70.6%, respectively. Likewise, the number of missing teeth increased. The overall average of 0.9 missing teeth per patient was subdivided in 0.48, 1.08 and 1.24 in missing teeth per patient in the CLA, UCLP and BCLP groups, respectively. Left-sided clefts were statistically significant more often affected than right-sided clefts (56.7% vs. 25.8%) (p = 0.006). In unilateral clefts missing teeth were more commonly located on the cleft side with a statistically significant difference to its contralateral side [51 teeth versus 15 teeth] (p << 0.001). For more details on the distribution of missing teeth per patient by tooth type and cleft type, see Table 3.

**Table 3:** Distribution of hypodontia dependent on cleft type and tooth type (absolute and percentage values)

		I2	P4	P5	Mandible	Total	
Cleft type	n	Absolute (%)	Absolute (%)	Absolute (%)	Absolute (%)	Absolute	Average missing teeth per person (absolute)
CLA	29	10 (17.4%)	0 (0.0%)	2 (3.4%)	2 (0.5%)	14	0.48
UCLP	62	27 (21.8%)	4 (3.2%)	23 (18.5%)	13 (1.5%)	67	1.08
BCLP	17	13 (38.2%)	1 (2.9%)	6 (17.6%)	1 (0.4%)	21	1.24
total	108	50 (23.1%)	5 (2.3%)	31 (14.4%)	16 (1.1%)	102	0.94

I2, lateral upper incisor; P4, first upper premolar; P5, second upper premolar; CLA, cleft lip and alveolus; UCLP, unilateral cleft lip and palate; BCLP, bilateral cleft lip and palate

### Supernumerary teeth

The cleft sample showed 33.3% supernumerary teeth (47 supernumerary teeth in 36 out of 108 patients). The female group presented ten out of thirty-nine female patients (25.6%), and the male group presented twenty-six out of sixty-nine male patients (37.7%) affected by supernumerary teeth, but a gender related statistical significance could not be found (p = 0.202). Generally, the maxillary lateral incisor was the most affected tooth (17.6%), but supernumerary teeth also affected the maxillary central incisor (1.9%) and mesiodentes as well (0.9%). In the mandible, supernumerary teeth could not be found in any case.

Concerning cleft type, individuals with a malformation of the primary palate solely (CLA), were significantly more often affected by supernumerary teeth than patients with a cleft of the lip and the palate (CLP) (p = 0.014). In CLA patients in 51.7% supernumerary teeth could be found, but only 29.0% and 17.6% of patients with UCLP and BCLP were diagnosed with supernumerary teeth, respectively.

There was a significant association between supernumerary teeth and the side of the cleft in the UCLP group (p = 0.035). In 45.5% of UCLP patients with a right-sided cleft supernumerary teeth could be found, but only 20.0% of UCLP patients with a left-sided cleft were affected. In the CLA group both cleft sides were equally affected by supernumerary teeth (see Table 4). Moreover, supernumerary teeth were most frequently located in the cleft area. Out of 41 supernumerary teeth found in unilateral clefts, 35 (85.4%) were located in the cleft area and only six (14.6%) on its contralateral side (p << 0.001). For more details on the distribution of supernumerary teeth by tooth type and cleft type, see Table 5.

**Table 4:** Distribution of patients with or without supernumerary teeth dependent on cleft type and side (absolute and percentage values)

Cleft side	CLA			UCLP		BCLP
	Right	Left	Bilateral	Right	Left	Bilateral
With supernumerary teeth	5 (55.6%)	10 (50.0%)	0	10 (45.5%)	8 (20.0%)	3 (17.6%)
Without supernumerary teeth	4 (44.4%)	10 (50.0%)	0	12 (54.5%)	32 (80.0%)	14 (82.4%)
Total	9	20	0	22	40	17

CLA, cleft lip and alveolus; UCLP, unilateral cleft lip and palate; BCLP, bilateral cleft lip and palate

**Table 5:** Distribution of supernumerary teeth dependent on cleft type and cleft side (absolute and percentage values)

	n	I2		I1		M	Total	
		Ipsilateral	Contralateral	Ipsilateral	Contralateral		Absolute	Average supernumerary teeth per person (absolute)
CLA	29	14 (48.3%)	1 (3.4%)	0 (0.0%)	1 (3.4%)	1 (3.4%)	17	0.59
UCLP	62	19 (30.6%)	3 (4.8%)	2 (3.2%)	0 (0.0%)	0 (0.0%)	24	0.39
BCLP	17	5 (29.4%)	–	1 (5.9%)	–	0 (0.0%)	6	0.35
Total	108	38 (35.2%)	4 (3.7%)	3 (2.8%)	1 (0.9%)	1 (0.9%)	47	0.40

I2, lateral upper incisor; I1, central upper incisor; M, mesiodens; CLA, cleft lip and alveolus; UCLP, unilateral cleft lip and palate; BCLP, bilateral cleft lip and palate

## Discussion

### Hypodontia

**Fig. 1:**
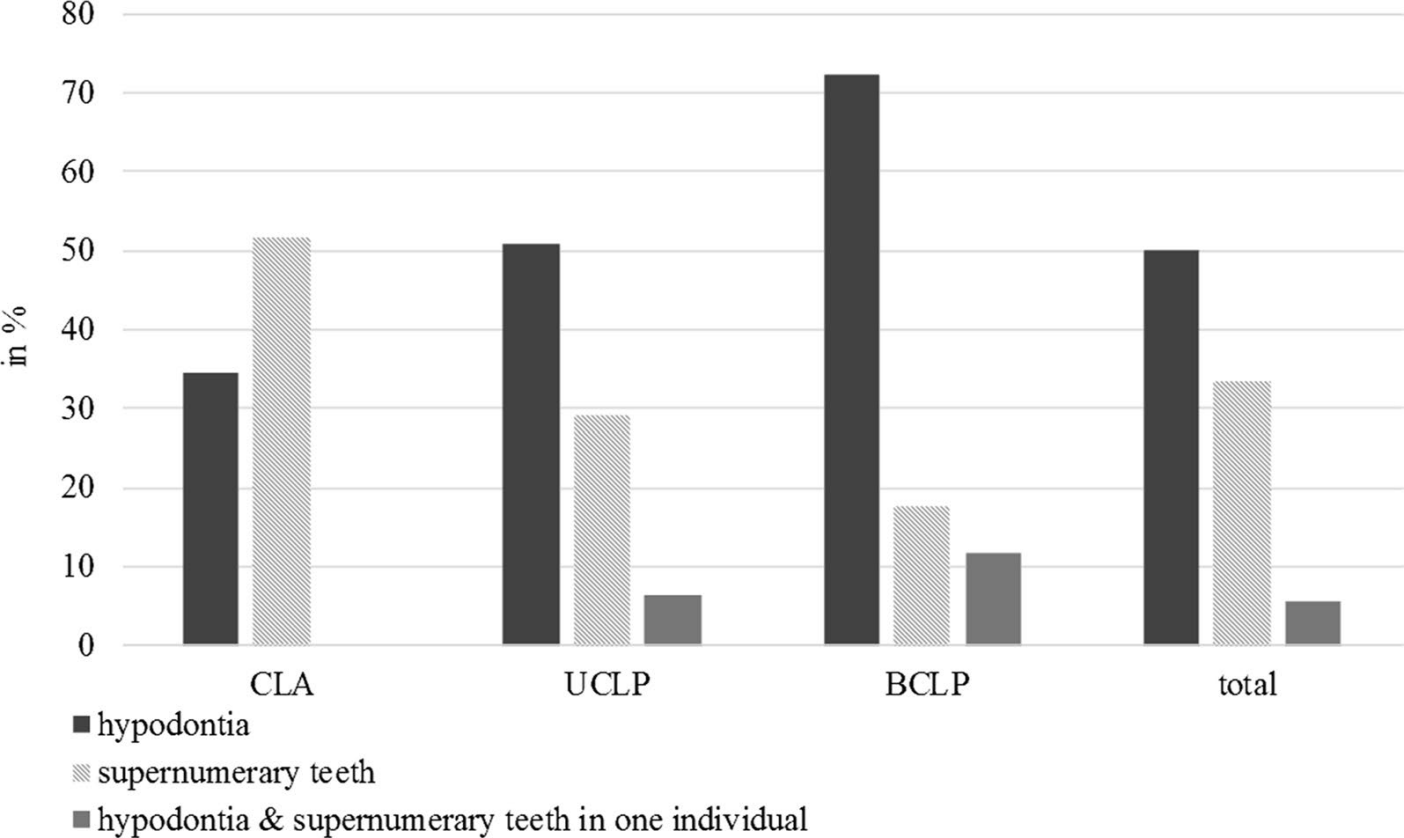
Prevalence of hypodontia and supernumerary teeth dependent on cleft type (values are illustrated in percentage of relevant cleft type).

Patients with craniofacial clefts are often affected by various dental anomalies, such as tooth agenesis, supernumerary teeth, microdontia, taurodontism, dilaceration, ectopic eruption, impacted teeth and late dental development [11, 19]. Among these, hypodontia is the most common one. In our investigation, 50% of the cleft patients analyzed, had congenital missing teeth. This is just a little less than the 62–73% reported in comparable studies [7, 11, 13, 20]. In contrast, one study reported an even lower prevalence of 38.6% for hypodontia [21]. These differences might be influenced by the composition of the cleft population analyzed or the relatively small sample size of the groups studied. Nevertheless, the prevalence found in our examination was still ten times of the prevalence of hypodontia calculated for the permanent dentition of a healthy European population, excluding third molars (5.5%) [10]. On the one hand, the order of the prevalence of absent teeth from highest to lowest, starting with the lateral upper incisor, followed by the upper second premolar and the lower second premolar, equates to those found in other studies [7, 11, 13, 20, 22], on the other hand, some authors calculated higher percentages for agenesis of the lower second premolars than the upper ones [5, 15, 23]. We found the upper lateral incisor to be the most commonly missing tooth in cleft patients with a prevalence of 23.2%, which is in agreement with other studies reporting percentages ranging in between 20 and 28% [7, 15]. However, in the literature, for the lateral incisor, there are also reports on a prevalence of hypodontia in cleft patients ranging in between 35% and 45% [11, 13, 20, 23]. In our study hypodontia of the upper second premolar was higher (14.2%) than values found in other studies, ranging from 5.3% to 10.4% [7, 13, 20, 23, 24]. Only one other comparable study showed higher values of 20.7% [11]. This inconsistency may be caused by small sample sizes, different composition of the cleft groups or even the time and type of cleft palate operation, as this could be an important environmental influence on hypodontia in cleft patients [8]. In the mandible in our study 5.1% of second premolars were missing, which is close to data reported for a healthy European population (2.9%–3.1%) [10] and studies on cleft patients with a comparable prevalence of 1.9%–3.5% found in their investigations [7, 13]. Otherwise, hypodontia of lower premolars has been described to be 6.6%–10.3% as well [11, 20, 23, 24]. We observed an increase of the frequency of hypodontia alongside with the extend of the cleft, which is in agreement with other investigations [6, 13] (see Fig. 1), as dental disorders in cleft patients usually increase with the severity of the cleft, thus the continuity of alveolar bone tissue harboring the tooth buds becomes more susceptible to dental alterations [27]. CLA patients of our collective were affected by hypodontia in 34.5%, UCLP patients in 51.6% and BCLP patients in 70.6%. In the CLA group, hypodontia was mainly caused by missing upper lateral incisors (17.2%), whereat in patients with an UCLP, in addition to the lateral incisors (21.8%), second premolars were missing (18.6%), too. In the BCLP group, second premolars were almost as affected by hypodontia (17.7%) as in the UCLP group, but the lateral incisors were missing 2.1 times more frequently (38.2%), explaining the high values of hypodontia found in that group. In the literature, the percentage and distribution of missing second premolars in cleft individuals showed a high variety, ranging from 0.0% [24] to 4.5% [11] for CLA, 6.2% to 33.0% for UCLP [24–26] and 10.8% to 28.5% for BCLP patients, respectively [11, 24–26]. Those differences could also be explained by different sample size and composition of the cleft population analyzed, as well as ethnical differences.

### Supernumerary teeth

In our study, supernumerary teeth were observed in 33.3% of cleft individuals. This result is much higher, compared to both, the calculated prevalence of supernumerary teeth in the permanent dentition of a healthy European population (1.0–2.2%) [14], and outcomes, previously reported for CL/P samples ranging in between 4.8% and 10.9% [7, 11, 13, 20, 23], but was very similar to the findings of Stahl et al., who found a prevalence of 32.2% for supernumerary teeth in the deciduous and permanent dentition of German cleft patients [6]. Our investigation revealed that in the UCLP group, right-sided clefts were statistically significant more often affected by supernumerary teeth than left-sided clefts, which is in accordance with the findings of Stahl et al., Byloff-Clar and Droschl [6, 11]. However, other studies did not confirm that right-sided clefts showed a higher risk of supernumerary teeth [7, 20]. In our cases, all supernumerary teeth found, were located in the maxillary anterior region. This is consistent with many other studies [7, 11, 13, 20, 23]. Except this, only Stahl et al. also found a supernumerary lateral incisor in the lower jaw [6]. However, in our investigation the maxillary lateral incisor was most often affected with a prevalence of 17.6%. Although these findings are higher than reports of other investigations with values of 5.9% and 12.7%, respectively [6, 11], they illustrate that in cleft patients the lateral incisor is the most frequent supernumerary tooth, followed by the central upper incisor and mesiodentes. Reports of affected upper canines can be found in the literature as well [11], but these results could not be confirmed within our study. Concerning cleft type, statistically significant differences of the prevalence of supernumerary teeth could be found in CLA patients (51.7%) and CLP patients (26.6%) (p = 0.014) (Fig. 1), which is in line with other reports of a higher prevalence for supernumerary teeth in CLA than in CLP patients [6, 7]. This could be attributed to the extend of the cleft and its effect on the epithelium, forming the dental germs. If a smaller extension of the cleft stops the epithelium from uniting, causing a supernumerary tooth, a larger cleft could cause microdontia or an even greater lack of epithelium, hypodontia [7]. That would explain the increasing prevalence of hypodontia and decreasing the prevalence of supernumerary teeth in CLP patients (Fig. 1). Interestingly Byloff-Clar and Droschl could not find such a difference [11]. Their survey on Austrian cleft patients demonstrated an equal distribution of supernumerary teeth throughout the three cleft groups with a prevalence of 9.1% for CLA, 10.9% for UCLP and 12% for BCLP patients. On the one hand, this variety in results shows the value of local data for treatment planning by surgeons and orthodontist, those dental disciplines deciding about balanced tooth extraction or gap opening for later implantation or when and which tooth to extract in case of supernumerary, not to harm other developing tooth germs. On the other hand, studies with a greater sample size would help to gain more information and a general view on the prevalence of numerical alterations in CL/P individuals. Actual data show, that pre-surgical orthodontic treatment will strongly improve bone healing after grafting and alveolar cleft repair [28]. Therefore, prevalence and location of numerical tooth alterations is very important to raise the awareness of good time to manage local dental problems in growing cleft patients.

Despite some limitations (small sample size of cleft patients and not including a non-cleft German control group, as it is difficult to include representative healthy individuals due to x-ray regulations and ethical concerns), this study helps to fill gaps in the current literature on local data on dental anomalies of German CL/P patients. For a general prevalence of hypodontia and supernumerary teeth in German cleft patients, we recommend a multi-center study. A greater sample size will help to get a clear picture of correlations between tooth count anomalies and cleft types, which might vary in small samples sizes due to different sample compositions and regional varieties.

## Conclusion

Based on the results gained in this survey, we conclude that:

- Hypodontia affects both, the maxillary and mandibular dentition
- Hypodontia increases with the extend of the cleft while the prevalence of supernumerary teeth decreases
- Supernumerary teeth, above all, can be found in the anterior region of the maxilla
- Right-sided clefts of UCLP patients are more susceptible to supernumerary teeth, while left-sided clefts of that type are more often affected by hypodontia
- Usually, numerical alterations are located in the cleft region itself, however, they can occur contralateral as well, but in a lower frequency

Therapists of cleft patients need to be aware of the high variety of numerical alterations, as this is important for therapy planning and the applied treatment.

## Data Availability

The datasets used and/or analysed during the current study are available from the corresponding author on reasonable request.

## Abbreviations

BCLP: Bilateral cleft lip and palate
CLA: Cleft lip and alveolus
CLP: Cleft lip and palate
CL/P: Cleft lip and alveolus with or without palate
UCLP: Unilateral cleft lip and palate
